# Occurrence and Treatment of Bone Atrophic Non-Unions Investigated by an Integrative Approach

**DOI:** 10.1371/journal.pcbi.1000915

**Published:** 2010-09-02

**Authors:** Liesbet Geris, Anita A. C. Reed, Jos Vander Sloten, A. Hamish R. W. Simpson, Hans Van Oosterwyck

**Affiliations:** 1 Division of Biomechanics and Engineering Design, Department of Mechanical Engineering, Katholieke Universiteit Leuven, Leuven, Belgium; 2 Prometheus, Division of Skeletal Tissue Engineering, Katholieke Universiteit Leuven, Leuven, Belgium; 3 Biomechanics Research Unit, University of Liège, Liège, Belgium; 4 Nuffield Department of Orthopaedic Surgery, University of Oxford, Oxford, United Kingdom; 5 Nuffield Department of Clinical Medicine, University of Oxford, Oxford, United Kingdom; 6 Department of Trauma & Orthopaedics, Edinburgh University, Edinburgh, United Kingdom; University of California San Diego, United States of America

## Abstract

Recently developed atrophic non-union models are a good representation of the clinical situation in which many non-unions develop. Based on previous experimental studies with these atrophic non-union models, it was hypothesized that in order to obtain successful fracture healing, blood vessels, growth factors, and (proliferative) precursor cells all need to be present in the callus at the same time. This study uses a combined *in vivo*-*in silico* approach to investigate these different aspects (vasculature, growth factors, cell proliferation). The mathematical model, initially developed for the study of normal fracture healing, is able to capture essential aspects of the *in vivo* atrophic non-union model despite a number of deviations that are mainly due to simplifications in the *in silico* model. The mathematical model is subsequently used to test possible treatment strategies for atrophic non-unions (i.e. cell transplant at post-osteotomy, week 3). Preliminary *in vivo* experiments corroborate the numerical predictions. Finally, the mathematical model is applied to explain experimental observations and identify potentially crucial steps in the treatments and can thereby be used to optimize experimental and clinical studies in this area. This study demonstrates the potential of the combined *in silico-in vivo* approach and its clinical implications for the early treatment of patients with problematic fractures.

## Author Summary

In light of the ageing population, the occurrence of bone fractures is expected to rise substantially in the near future. In 5 to 10% of these cases, the healing process does not succeed in repairing the bone, leading to the formation of delayed unions or even non-unions. In this study we used a combination of an animal model mimicking a clinical non-union situation and a mathematical model developed for normal fracture healing to investigate both the causes of non-union formation and potential therapeutic strategies that can be applied to restart the healing process. After showing that the mathematical model is able to simulate key aspects of the non-union formation, we have used it to investigate several treatment strategies. One of these strategies, the treatment of a non-union involving a transplantation of cells from the bone marrow to the fracture site, was also tested in a pilot animal experiment. Both the simulations and the experiments showed the formation of a bony union between the fractured bone ends. In addition, we used the mathematical model to explain some unexpected experimental observations. This study demonstrates the added value of using a combination of mathematical modelling and experimental research as well the potential of using cell transplantation for the treatment of non-unions.

## Introduction

Atrophic non-unions, a class of non-healing fractures that display only limited external callus formation, were thought to occur as a result of impaired local blood supply [1], [2]. However, studies of human atrophic non-unions have shown that the gap tissues can be well vascularized [3]–[5]. Recently several animal models were developed to investigate the etiology of atrophic non-unions [6]–[9]. In these animal models, the periosteum disruption and reaming of the marrow canal is combined with adequate stabilization of the osteotomy site. All models demonstrate established non-unions. These animal models are a good representation of the clinical situation in which most atrophic non-unions develop [8]. Previous non-union models often utilized large segmental bone defects [10]–[13] where non-unions developed due to the size of the defect rather than the altered biology of the fracture site [8].

Based on previous, purely experimental, studies with these atrophic non-union models, it was hypothesized that in order to obtain successful fracture healing, blood vessels, growth factors and (proliferative) precursor cells all need to be present in the callus at the same time [6], [7]. In this study we will use a combined *in vivo*-*in silico* approach to look at these different aspects (vasculature, growth factors, cell proliferation). Furthermore, the *in silico* model is used to investigate the occurrence of and design possible treatment strategies for atrophic non-unions. Experimental results (original work and previously published) are used to corroborate the numerical predictions for atrophic non-unions in particular and demonstrate the potential of both the *in silico*-*in vivo* approach and the treatment strategy of cell transplantation. Finally, the mathematical model is applied to explain experimental observations and identify potentially crucial steps in the treatments.

## Materials and Methods

### Ethics statement

Rats were kept in accordance with UK Home Office welfare guidelines and project license restrictions.

### Animals and operative procedure

Animals and operative procedures were carried out as previously reported [7]. In brief, 28 adult female Wistar rats were randomised into 2 groups of 14 (‘non-union’ and ‘healing’) and were sacrificed at 1 (n = 3), 3 (n = 3), 8 (n = 4) and 16 (n = 4) weeks and the right (operated) tibia was prepared for histological examination. The animals were caged individually and allowed water and food *ad libitum* and unrestricted weight-bearing.

A standardised circular frame external fixator [7] was applied to the right tibia under general anaesthesia and with aseptic conditions. An osteotomy was performed using a 1mm burr under constant irrigation with cold saline solution. The fibula was fractured manually using a three-point bending method and a 1 mm gap introduced at the site of the osteotomy. In 14 of the 28 animals the periosteum was stripped and the intramedullary canal was curetted, both proximally and distally, for a distance equivalent to 1 tibial diameter. The wound was washed thoroughly and the skin was closed.

Two independent senior orthopaedic trainees assessed standardized radiographs obtained after operation and every two weeks thereafter. They categorized the fractures as healing or not according to the criteria of the AO-ASIF manual [2].

### Cell transplant

Atrophic non-union was induced in 8 WKY rats as previously described [7]. Three weeks after operation, 4 rats received a 100 µl injection at the non-union site of cultured bone marrow cells and 4 rats received a 100 µl injection of carrier solution alone. Bone marrow cells were obtained by aspiration of the hind limbs of WKY rats and processed for injection as described in (a full description of this protocol is submitted for publication elsewhere). Two oblique radiographs were taken of the right (operated) tibia post-operatively and at 2 weeks, 3 weeks (immediately following cell/carrier injection) and every week thereafter. Radiographs were examined by two independent senior orthopaedic trainees. Each animal was categorized as healing or not (full report of results submitted for publication elsewhere).

### Measurement of callus formation

Formation of callus was assessed by scanning radiographs into a Macintosh Quadra 650 computer and analyzing images (Optilab Pro v2.5, Graftek, France). The callus outline was traced manually and the size of the outlined area was calculated. The results were expressed as a percentage change in the amount of mineralized tissue from post-operative radiographs [7].

### Histology and immunohistochemistry

The right lower limbs were fixed in neutral phosphate-buffered formalin (4% v/v) for 48 hours, decalcified in neutral ethylenediaminotetra-acetic acid (EDTA), embedded in paraffin wax and 6µm sections were cut and stained with haematoxylin and eosin. Wax sections for immunohistochemistry were cut onto poly-l-lysine coated slides and immunostaining was performed with the following antibodies: Transforming growth factor beta (TGF-β) (mouse monoclonal, AbD Serotec Ltd, Oxford, UK), Basic Fibroblast Growth Factor (FGF basic) (goat polyclonal, Santa Cruz Biotechnology Inc. USA), Platelet-Derived Growth Factor (PDGF) (goat polyclonal, R&D Systems Ltd, Abingdon, UK), Bone Morphogenetic Proteins 2 and 4 (BMP 2/4) (goat polyclonal, R&D Systems Ltd, Abingdon, UK), and Proliferating Cell Nuclear Antigen (PCNA) (mouse monoclonal, Dako Ltd, Ely, UK). Alkaline phosphatase-conjugated anti-mouse (Dako, Ely, UK) or anti-goat (Sigma, Poole, UK) secondary antibodies were used. Cell transplant samples labelled with BrdU were prepared as above, followed by immunostaining with antibodies against BrdU (mouse monoclonal, Dako Ltd, Ely, UK) in conjunction with an animal research kit (ARK, Dako Ltd, Ely, UK). All antibodies were used according to the manufacturer's instructions.

Sections were analyzed using a light microscope. Cytoplasmic growth factor expression was semi-quantified using a four-value intensity score (0, 1+, 2+, and 3+) [14]. For proliferating cells, the numbers of positively and negatively stained cells were counted in randomly selected fields within the interfragmentary gap and the median positive staining index was calculated.

### Statistics

A Mann-Whitney U test was used to assess significance of the cell proliferation results. All statistical analyses were performed using the Statview software package (SAS Institute Inc., USA) and significance was assumed as p<0.05.

### Mathematical modelling

**Figure 1:**
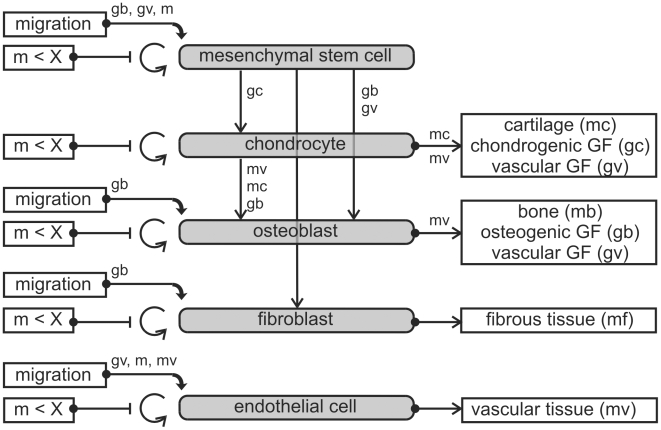
Schematic representation of the mathematical model. Legend: GF = growth factor, m = mf + mc + mb + mv = total tissue density, X = maximum tissue density for proliferation. The involvement of a variable in a regeneration subprocess is indicated by showing the name of that variable next to the arrow representing that particular process, e.g. the vascular matrix density (mv) interferes with cell migration, endochondral ossification and GF production.

The mathematical model used in this study was originally developed to describe normal fracture healing [15]. It expresses the change of a number of continuum-type variables – growth factor concentrations, cell densities and matrix densities – as a function of time and spatial coordinates and is schematically represented in Figure 1. The model accounts for various key processes of bone regeneration. Starting with a callus filled with granulation tissue, mesenchymal stem cells and growth factors quickly occupy the regeneration zone. This is followed by mesenchymal stem cell differentiation into osteoblasts (intramembranous ossification – close to the cortex away from the fracture site) and chondrocytes (central callus region). Subsequently, endochondral ossification can take place during which VEGF, expressed by (hypertrophic) chondrocytes, attracts blood vessels and osteoblasts, resulting in cartilage degradation and bone formation. Bone remodelling processes are not included in the model. The effect of mechanical loading can also be incorporated, by making various biological processes dependent on local mechanical stimuli [16]. Mechanical influences will however not be the subject of the current study.

The regeneration processes are described by calculating the spatiotemporal evolution of the density of mesenchymal stem cells, osteoblasts, chondrocytes, fibroblasts, endothelial cells, bone, cartilage, fibrous tissue and vascular matrix and the concentrations of three generic growth factor families (osteogenic, chondrogenic and vascular growth factors). The spatiotemporal dynamics is expressed by means of a system of 12 partial differential equations of the taxis-diffusion-reaction type. The system's general structure is:
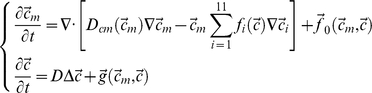
(1)t represents the time, 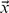 the space and 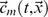 the non-dimensional density of a migrating cell type (mesenchymal stem cells, fibroblasts and endothelial cells). 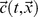 represents the vector of the other nine non-dimensional cell concentrations, ECM densities and growth factor concentrations (for which no directed migration is modelled). 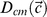 and *D* (non-negative diagonal matrix) are the diffusion coefficients (random motion). 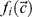 represents the taxis coefficients for both chemotaxis (movement up a gradient of growth factor concentration) and haptotaxis (movement up a gradient of matrix density). 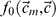 and 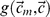 are reaction terms describing cell differentiation, proliferation and decay and matrix and growth factor production and decay. The system (1) must be complemented by suitable initial and boundary conditions to ensure the existence, uniqueness and non-negativity of a solution 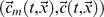. The model equations are implemented in a customized finite volume code [17]. Additional information, including the complete set of equations, boundary and initial conditions, parameter values and implementation details, is provided in the Supplementary Methods (Text S1) and Geris *et al.* [15]. For a detailed discussion of the model's underlying assumptions, simplifications and shortcomings, we refer the reader to Geris *et al.* [15], [16]. Non-union cases were previously simulated by compromising the initial blood vessel or growth factor supply [15] or by applying increased mechanical loading (instability) on the fracture [16]. In all cases, the simulations were able to capture observed experimental and clinical outcomes. Moreover, *in silico* experiments were conducted to design potential treatment strategies for the various non-union models [15], [18], [19] however, these predictions were not corroborated by dedicated experiments as is the case in this study.

**Figure 2:**
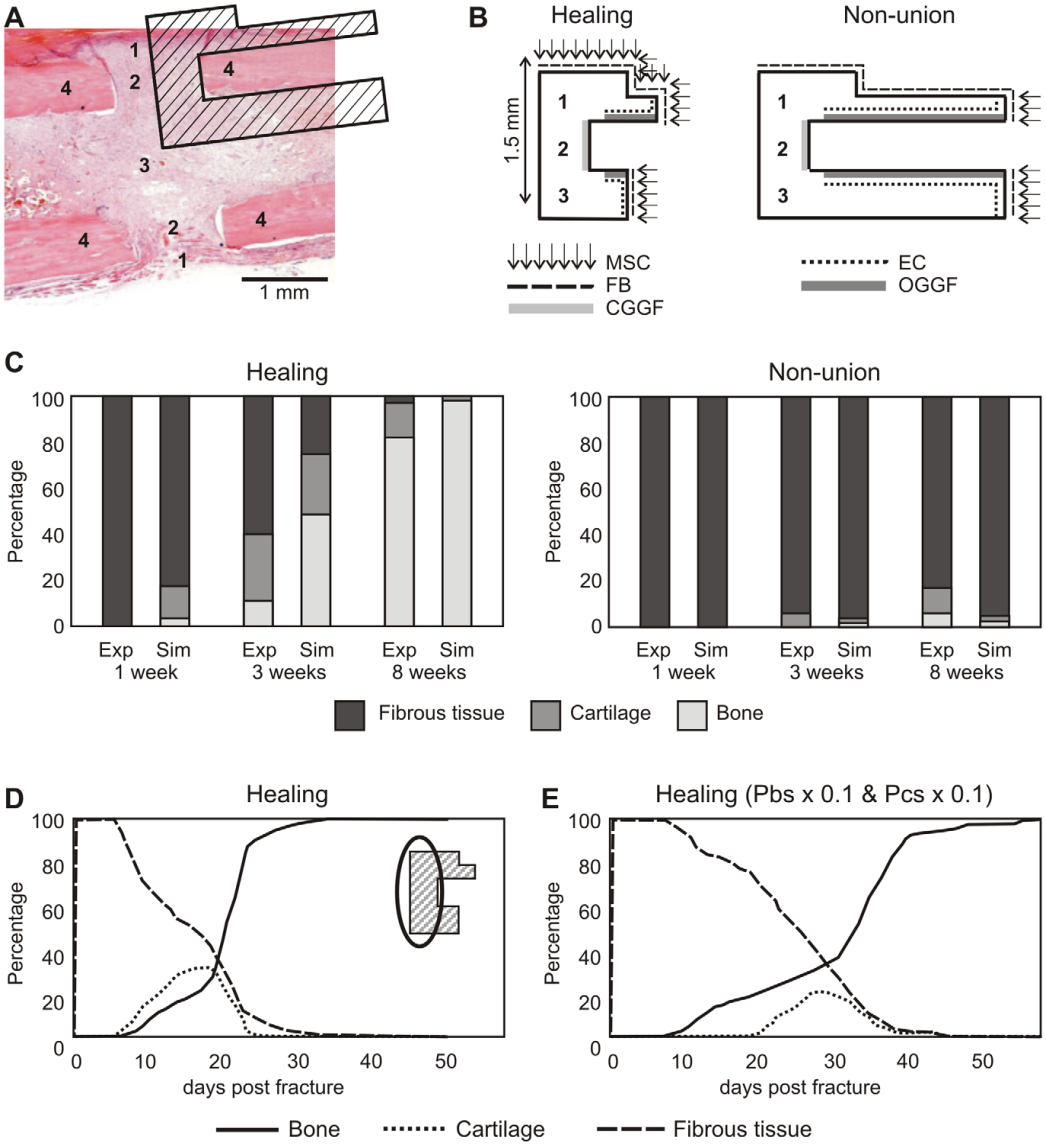
Mathematical modelling of the regeneration process in healing and non-union groups. (**A**) Domain of the simulations. 1: periosteal callus; 2: intercortical gap; 3: endosteal callus; 4: cortical bone. (**B**) Boundary conditions for healing and non-union model. MSC: mesenchymal stem cells; FB: fibroblast; OB: osteoblast; EC: endothelial cell; CGGF: chondrogenic growth factor; OGGF: osteogenic growth factor. (**C**) A comparison of experimentally measured (Exp) [7] and numerically calculated (Sim) tissue constituents present within the interfragmentary gap of healing and non-union groups (stacked bars). (**D**) Temporal evolution of the numerically calculated tissue fraction for the healing group. The insert shows the interfragmentary region of the simulation domain. (**E**) Temporal evolution of the numerically calculated tissue fraction for the healing group with reduced (by factor 10) cartilage (P_cs_) and bone matrix (P_bs_) production rates leading to a better correspondence between experimental and simulation results.

A simplified (fixed) geometrical domain of a fracture callus (Fig. 2A,B) was constructed based on the experimental set-up [7]. For the atrophic non-union case the domain was extended at the distal end (away from the fracture site) over the distance that the periosteum was stripped and the marrow canal was reamed in the experiments. The current implementation of the model assumes a constant callus size for both healing and non-union groups (Fig. 2B). The experimentally observed callus size for both healing and non-union groups is described in [7]. For the non-union group there was a trend towards a decrease of the callus-size (by 10%) over time, though this was not a statistically significant decrease.

It was shown on a rabbit atrophic non-union model [6] that the (lack of) granulation tissue containing the necessary MSCs plays an important role in the formation of a non-union. Therefore, in order to simulate the formation of an atrophic non-union, the boundary condition for the MSCs was adapted (value of the Dirichlet boundary conditions was decreased with a factor 10^5^) while all other model parameters and initial/boundary conditions were left unchanged with respect to the normal healing case. As the initial mechanical conditions for both the healing and non-union group were equal, the influence of mechanical loading was not taken into account explicitly in this study (the bioregulatory model presented in [15] was used rather than an extended mechanobioregulatory model presented in [16]). After initial analyses, the values for the growth factor boundary conditions (which were estimated in [15]) were lowered by a factor 10 in this study (for both the healing and non-union case) to obtain a better correspondence with experimental results (for both healing and non-union case). This alteration only affected the average growth factor concentration during the first weeks of the healing process but had no effect on the amount and distribution of the cells and extracellular matrix. For all simulations cartilage and bone matrix formation rates were equal to the values reported in [15], apart from a case in which these values were lowered by a factor 10. This was done in order to investigate the effect on the healing rate. For all the simulated treatment strategies, 1ml of MSCs was administered at a concentration of 10^6^ cells/ml.

Tissue fractions were calculated from the bone, cartilage and fibrous tissue matrix densities in the central area of the domain, corresponding to the experimentally investigated region of interest (i.e. excluding those parts of the domain stretching out to the right alongside the cortex, see insert in Fig. 2D).

## Results

When simulating the healing process on a fracture where either the periosteum was stripped or the marrow canal was reamed, complete fracture healing was predicted to occur with delayed bone formation in those parts of the callus domain where the MSC source was removed. Only when both modifications were combined (Fig. 2A,B) the occurrence of a non-union was predicted (Fig. 2C), suggesting that, from a modelling perspective, lack of primitive cells due to the stripping of the periosteum and the reaming of the intramedullary canal influences other processes further downstream in the healing process, such as blood vessel formation and growth factor production, leading eventually to a non-union.

### *In silico* and *in vivo* atrophic non-union model

The simulation results showed healing progression similar to experimental results [7], for both the healing and the non-union group (Fig. 2C). For the non-union group, the mathematical model predicted the formation of small amounts of cartilage and bone by post-osteotomy week (POW) 8. For the healing group, both direct bone formation (close to the undamaged cortical bone) and cartilage formation (central part of the callus) were predicted to form by POW3 (Fig. 2D). By week 8 this cartilage was replaced by bone via endochondral ossification in the simulations. Reducing the cartilage and/or bone matrix formation rate in the mathematical model resulted in a slower ossification process (Fig. 2E).

**Figure 3:**
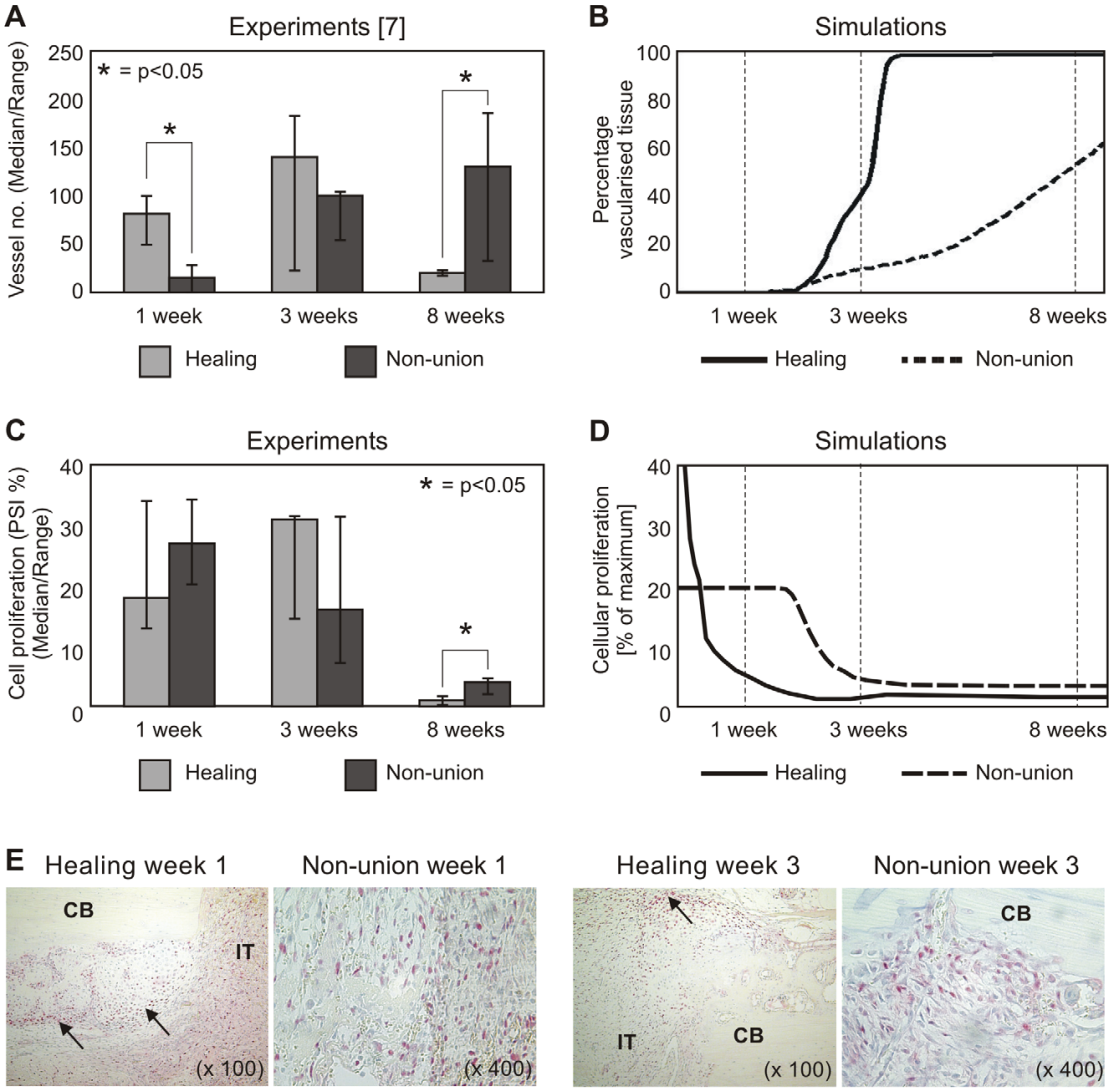
Vascularisation and cell proliferation in healing and non-union groups. (**A**) Comparison of the median number of blood vessels between the healing and non-union groups [7]. (**B**) Percentage of vascularised tissue in the callus as calculated by the mathematical model. (**C**) A comparison of the number of PCNA positive cells within the interfragmentary gap at 1, 3, 8 and 16 weeks post-osteotomy between healing and non-union groups. (**D**) Cellular proliferation for the healing and non-union set-ups, as calculated by the mathematical model. (**E**) PCNA expression indicating cell proliferation in the fracture area. For the healing group PCNA expression is found along the edge of callus formation in the periosteum (arrows) and within the interfragmentary tissue (IT) at POW1 (magnification ×100) and along the callus ossification front (arrow) and within the interfragmentary tissue at POW3 (magnification ×100). The non-union group shows PCNA stained cells in the interfragmentary gap at POW1 (magnification ×400) and PCNA positive cells surrounding areas of active bone resorption at POW3 (magnification ×400). CB = cortical bone.

As blood vessels are represented in the mathematical model by a continuum variable, the amount of vessels itself cannot be quantified from the simulation results. Instead, the percentage of vascularized tissue was calculated. Due to the absence of the vasculogenesis process (i.e. process of blood vessel formation occurring by a *de novo* production of endothelial cells in contrast to angiogenesis where blood vessels are formed from pre-existing ones [20]) in the mathematical model, blood vessel formation only appeared at the onset of osteogenesis (week 1) (Fig. 3A,B). By 8 weeks, the non-union group reached the level of vascularisation that was present in the healing group at 3 weeks (Fig. 3B).

In the experimental non-union group, cell proliferation was at its highest one-week post-osteotomy, with PCNA positive cells present throughout the interfragmentary gap. The number of PCNA positive cells within the interfragmentary gap diminished at 3 and 8 weeks post-osteotomy (Fig. 3C,E). In the healing group, cell proliferation peaked at week 3, where PCNA positive cells were present in the periosteum and at the edge of the ossification front (Fig. 3E). By 8 weeks the number of PCNA positive cells had diminished, and they were only evident in the periosteum at the periphery of the bridging callus.

In the numerical simulations, rapid proliferation of the MSCs at the onset of the healing process spiked the value for cellular proliferation in the first week for the healing group in contrast to the non-union group (Fig. 3D). After differentiation into chondrocytes in the central part of the callus and subsequently into osteoblasts, the cellular proliferation dropped in the healing group. Fibroblasts are the predominant cell type in the non-union group and as the predicted fibrous tissue density was not as high as that of bone or cartilage (leaving less space for cell proliferation) for the healing group, the non-union group was predicted to have a higher proliferative capacity at POW 8 compared to the healing group, corresponding to experimental *in vivo* observations.

**Figure 4:**
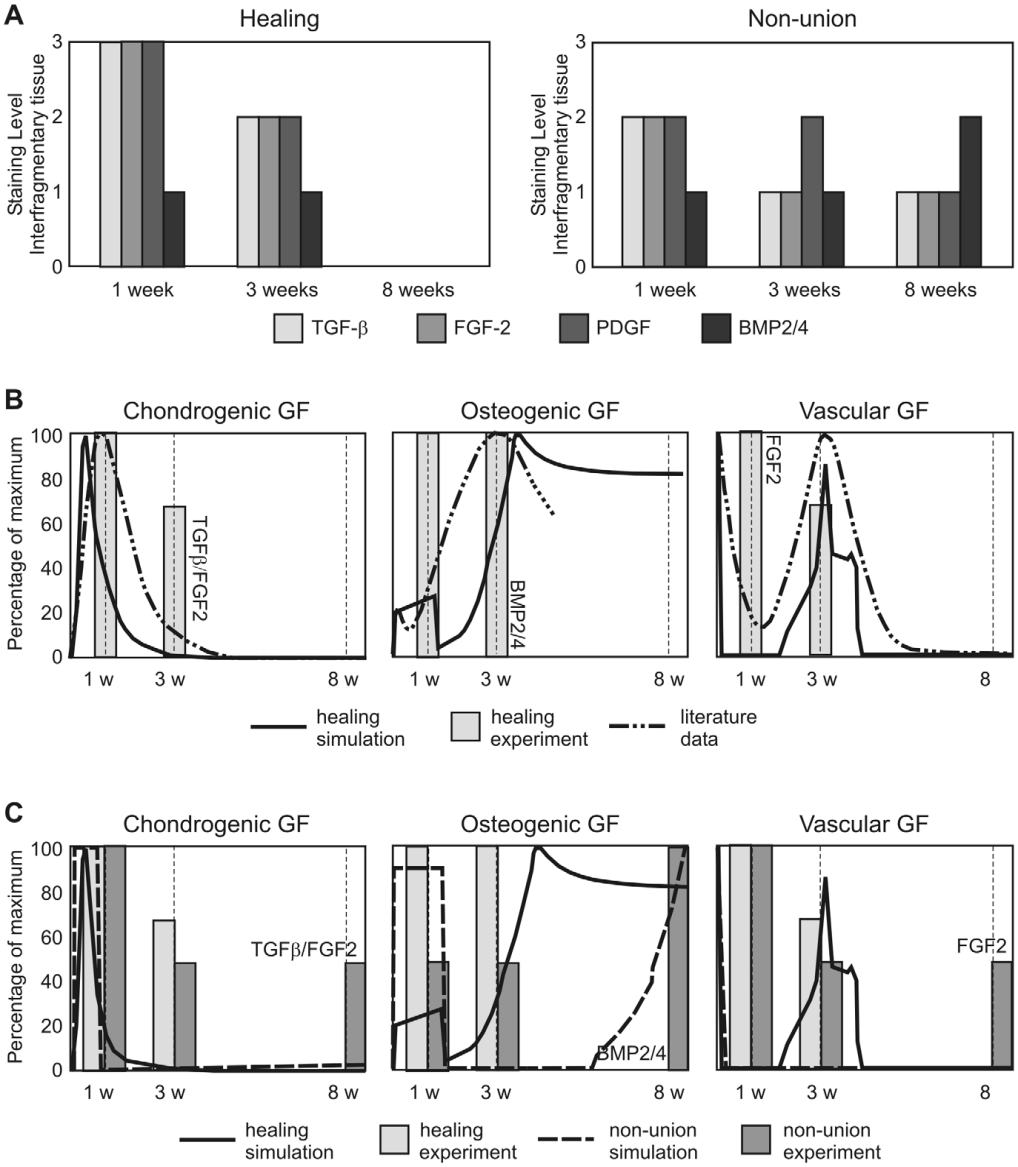
Growth factor expression in healing and non-union groups. (**A**) A comparison of growth factor staining index (based on immunohistochemical analysis) within the interfragmentary gap at 1, 3, 8 and 16 weeks post-osteotomy between healing and non-union groups. (**B**) Relative variation of the density of the generic osteogenic, chondrogenic and vascular growth factor density as calculated by the mathematical model for the healing group (full line) and reported in literature (dash-dotted line) [21]–[23]. Bars represent the experimentally measured growth factor levels of the growth factors belonging to these functional families (according to [21]–[23]) for the normal healing group. (**C**) Relative variation of the density of the generic osteogenic, chondrogenic and vascular growth factor density, as calculated by the mathematical model for healing and non-union set-ups. Experimentally measured (bars) growth factor variations of growth factors belonging to these functional families (according to [21]–[23]) are depicted as well.

At POW 1, the hematoma within the interfragmentary gap of the *in vivo* experimental healing and non-union groups stained positively for TGF-β, FGF-b, PDGF and BMP 2/4, however, TGF-β and FGF-b staining of the hematoma in the non-union group appeared weaker than that of the healing group (Fig. 4A and Figure S1). At POW 3, staining of all four growth factors was evident in areas of endochondral ossification in the healing group, where both osteoblasts and chondrocytes were expressing these growth factors. In the non-union group, however, TGF-β, FGF-b and BMP 2/4 staining had diminished in comparison to the one week time point. At 8 and 16 weeks (identical results were obtained for both time points), there was either weak or absent staining of all four growth factors in the healing group, due to bridging callus. In the non-union group, weak staining of all 4 growth factors remained in the fibrous tissue of the interfragmentary gap.

In the mathematical model, generic, functional families rather than specific growth factors were implemented. The average concentration for each generic growth factor group was calculated for the central area of the domain (indicated on insert in Fig. 2D). In Figure 4B, the experimentally measured growth factors for the healing group are depicted according to their classification in generic growth factor families by Cho *et al.* [21], Pepper *et al.* [22] and Lienau *et al.* [23]. Osteogenic growth factors are expressed early on in the healing process during intramembranous ossification. Later on, an increase in their production is predicted during the endochondral ossification process. For the vascular growth factors, after the initial decrease, upregulation is predicted during the endochondral ossification process taking place in the healing group, where VEGF is being expressed by (hypertrophic) chondrocytes. For both the healing and non-union groups, the highest levels of chondrogenic growth factors are predicted by the mathematical model in the first week, similar to the experimental measurements. For the non-union group (Fig. 4C), after the initial growth factor release at fracture induction, some chondrogenic growth factor production remains present up to and after POW 8. Osteogenic growth factor production is predicted to rise between POW3 and POW8 in the non-union group, as experimentally observed.

### Treatment strategies for atrophic non-unions

**Figure 5:**
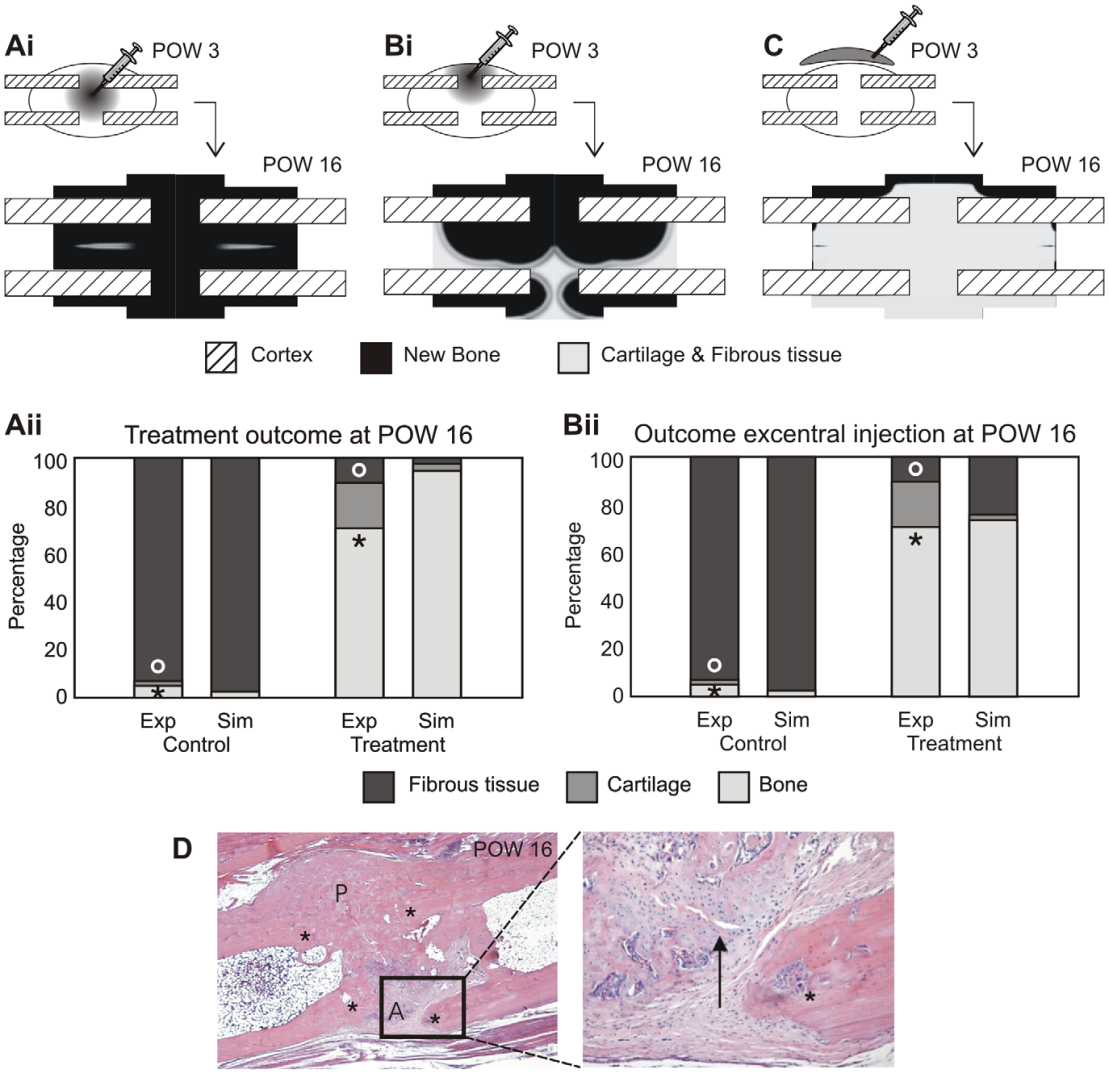
The effects of MSC transplantation on atrophic non-union. (**Ai**) Simulation results for the treatment with the cell transplant injected in the centre of the callus. (**Aii**) A comparison of experimentally measured (Exp) and numerically calculated (Sim) tissue constituents present within the interfragmentary gap of control (carrier solution injected) and treatment (MSC transplant) groups (^o^* p<0.005, students t-test). Simulation results are shown for a central injection of the carrier solution. (**Bi**) Simulation results for the treatment with the cell transplant injected excentrally in the callus. (**Bii**) A comparison of experimentally measured (Exp) and numerically calculated (Sim) tissue constituents present within the interfragmentary gap of control (carrier solution injected) and treatment (MSC transplant) groups (^o^* p<0.005, students t-test). Simulation results are shown for an excentral injection of the carrier solution. (**C**) Simulation results for the treatment with the cell transplant injected outside the callus. (**D**) Histological section (H&E) showing full bony callus bridging of the posterior (P) aspect of the tibia but no bony bridging at the anterior (A) aspect. Original bone ends marked *. An area of endochondral ossification (arrow) is present at the anterior aspect of the tibia. Images taken at ×20 magnification.

After corroboration of the mathematical model, we wanted to test, both *in silico* and *in vivo*, the hypothesis that the onset of atrophic non-union could be prevented by the injection of cultured MSCs at three weeks post-osteotomy, i.e. when vascularity within the interfragmentary gap was sufficient to keep the injected cells alive. *In silico*, after the injection of the cell transplant at POW 3 in the callus region, the amount of bone was predicted to gradually increase whereas the amount of fibrous tissue was predicted to decrease up to POW 16. The formation of a small amount of cartilage was also predicted with endochondral ossification still in progress at POW 16 (Fig. 5Ai,Aii). The amount of soft tissue present at POW 16 was strongly dependent on the exact location of injection of the cell transplant with excentral injection leading to unicortical bridging (Fig. 5Bi,Bii). A technique often used to administer growth factors to healing fractures is the administration of growth factors inside an injectable carrier close to but outside of the callus (reviewed in [24]). Alternatively, one could adopt such a carrier approach for cell delivery as well. However, simulating such a treatment strategy predicted the formation of a layer of bone closest to the cell source, preventing other cells from further penetrating the callus (Fig. 5c).

## Discussion

In the simulations, a combination of periosteal stripping and marrow canal curettage led to non-union formation, thereby predicting the observed *in vivo* outcome, where all animals that had periosteal stripping and curettage of the intramedullary canal went on to form an atrophic non-union at 8 and 16 weeks post-osteotomy and all animals where this was not performed went on to unite successfully [7]. At 1 week there was no significant difference in tissue constituents between the two experimental groups. However, from 3 weeks onwards, there was a significant increase in bone formation in the healing group when compared to the non-union group where the interfragmentary gap consisted predominantly of fibrous tissue. The model did not predict the experimentally observed rounding of the cortical bone ends in the non-union group, nor the capping of the intramedullary canal. The cortical bone falls outside of the modelling domain so changes in the cortical bone cannot be predicted by the model. The mechanism which causes the capping of the intramedullary canal is not known and does not seem to be encompassed by the present model equations either.

Although the order of fracture healing events in the simulations corresponds to experimental observations, the normal healing enrolled faster in the simulations than in the experiments [7]. As the predicted time frame of healing as well as the predicted tissue pattern correspond well to other rat fracture models [8], [25], we speculate that this difference might be attributed to a number of factors that are specific for this experimental set-up. The initial delay between experimental observations and numerical simulations is one week (Fig. 2D). This could be due to a particularly lengthy inflammatory phase in this specific experimental set-up, a phase that is not incorporated explicitly in the mathematical model. After POW 3, the endochondral ossification process enrolls much faster *in silico* than *in vivo* increasing the time delay between experiments and simulations. Decreasing the endochondral ossification speed *in silico* by e.g. reducing the cartilage and bone matrix production rate in the model leads to a better correspondence between experiments and simulations (Fig. 2E). For all other simulation results shown in this study the original values for cartilage and bone matrix production rates as determined in [15] were used.

Similar to the experimental observations [7], by 8 weeks, the non-union group reached the level of vascularisation that was present in the healing group at 3 weeks (Fig. 3B). The slower vascularisation of the simulated non-union with respect to its experimental counterpart could be due to the absence of *de novo* blood vessel formation (vasculogenesis) in the mathematical model combined with the absence of vascular growth factor production by fibroblasts, leading to a slow build-up of the vascular network. The experimentally observed decrease in number of blood vessels during bone/blood vessel remodelling at POW 8 (which is often accompanied by an increase in vessel diameter, a parameter that is not included in the experimental measurements) cannot be predicted as the model does not encompass the remodelling process. Despite these limitations, the mathematical model does predict a substantial time difference in the vascularisation of the callus area as shown by the (also experimentally observed) lag in blood vessel formation between healing and non-union groups at POW 3.

Cell proliferation observed in the *in vivo* healing group, concurred well with that observed by Iwaki *et al.* [26] where proliferating cells peaked between 1 and 3 weeks after fracture. For the proliferation, no exact match to the experimental measurement could be obtained from the simulation results. A general cellular proliferation value was calculated by multiplying the cells in the callus by their respective proliferation rates (which are fixed parameters in the mathematical model), normalized to total cell amount and the maximal proliferation rate. As all cells in the mathematical model are able to proliferate, providing there is sufficient space surrounding them (i.e. ECM density is sufficiently low), this calculated value is merely a theoretical one. The initial low density fibrous matrix present at the start of the healing simulations allows for rapid proliferation of the MSCs, spiking the value for cellular proliferation in the first week for the healing group in contrast to the non-union group where very few MSCs were present (Fig. 3D). The formation of differentiated tissue types such as bone and cartilage constrained the proliferation of the cells later on in the regeneration process for the healing group. As the fibrous matrix that develops in the non-union group does not reach the density of the bone or cartilage in the healing group, the, mainly fibroblastic, cells in the former group had a higher proliferative capacity at POW 8 compared to the latter, as observed *in vivo*.

Growth factor expression observed experimentally during the early stages of bone healing correlated well with other studies reporting BMP [27], TGF-β [28], and FGFb [28], [29] expression in normally healing rat fracture models, and PDGF expression in normal and impaired human fracture healing [30]. Furthermore, these results correlate with those of Brownlow *et al.* [6] who noted in a rabbit atrophic non-union model that by POW 8, there was little or no expression of TGF-β, FGFb, PDGF or BMP 2/4. Cho *et al.* [21], Pepper *et al.* [22] and Lienau *et al* [23] classified growth factors in functional families, similar to those used in the mathematical model. The reported evolution over time of these growth factor families corresponds well to the model predictions for the healing group (Fig. 4B). For the vascular growth factors, after the initial decrease, upregulation is predicted during the endochondral ossification process taking place in the healing group, where VEGF is being expressed by (hypertrophic) chondrocytes, corresponding well to previously reported experimental observations [31]. The experimentally observed decrease in osteogenic growth factors after week 3 is not present in the model as the osteoblasts remain active (i.e. keep producing growth factors). Addition of a supplementary variable representing the matured osteoblasts (osteocytes) could resolve this discrepancy between experiments and simulations. For the non-union group, the osteogenic growth factor levels are predicted to rise between POW3 and POW8, corresponding to experimental *in vivo* observations. Furthermore, the simulations show occurrence of the highest levels of chondrogenic and vascular growth factors in the first week, as experimentally observed. In the mathematical model chondrocytes are the only cell type capable of chondrogenic growth factor production and the main responsible for the production of vascular growth factor (upon hypertrophy). As chondrocytes are predicted to be only marginally present in the callus of the non-union group, the predicted chondrogenic and vascular growth factor concentrations further on in the healing process are very low for this group in comparison to the growth factor release at fracture induction. Furthermore, for the non-union group, initial growth factor concentrations are strongly driven by boundary conditions that were applied during the first days after fracture to mimic local and systemic reactions occurring outside of the modelling domain [32], [33].

Comparison of experimental and simulation results showed a number of discrepancies that could be attributed to a number of model simplifications and suggestions were made above to overcome those. Additional model simplifications were made in the framework of this study such as the use of a fixed callus size (rather than a size that varies over time) and the continuum representation of blood vessel formation, which will be dealt with in future versions of the mathematical model. For a thorough discussion on the model limitations we refer the reader to [15]. In this study, mechanical loading was not modelled explicitly as the initial mechanical situation was the same for both the healing and the non-union group. During the healing process, the development of stiffer tissues such as bone might alter the local mechanical conditions in the healing group thereby possibly influencing the healing process. However, under normal mechanical conditions (i.e. appropriate external stabilisation that allows for normal healing to occur) the bioregulatory model used in this study [15] behaves the same as an extended mechanobioregulatory variant [16] that incorporates mechanical loading explicitly.

The use of stem cells in the treatment of non-unions is gaining interest (reviewed in [34]). However, to date these stem cells were delivered in a (ceramic) scaffold or carrier structure requiring invasive surgery. Non-invasive techniques such as direct injection of a cell-buffer mixture or the use of injectable carriers could substantially reduce the additional trauma for the patient and were investigated by an *in silico – in vivo* approach in this study. As blood vessel formation is delayed in the non-union group, the most suitable time point for intervention of either growth factor treatment or cell transplant, seems to be three weeks post-osteotomy, when the blood supply to the interfragmentary gap has started to recover. Injection of MSCs directly into the callus area elicited to a good healing response *in silico*. Experimental results confirmed that transplantation of MSCs into the interfragmentary gap at POW 3 prevented the onset of an atrophic non-union. There was significantly more bone present in the treatment group (cell transplant) than in the control group (carrier solution). Union by bridging callus had occurred in three of the four treatment animals. Yet, all of the animals treated by MSC transplantation displayed an asymmetric healing with endochondral ossification in progress in part of the intercortical callus (Fig. 5D). As the zone of endochondral ossification was not always observed on the same aspect of the tibia (anterior vs posterior), mechanical loading was ruled out as the major cause. The *in silico* experiments carried out in the framework of this study identified another potential cause, namely injection of the cell transplant excentrally in the callus. Upon excentral injection at POW 3, cartilage undergoing endochondral ossification was predicted to be present at the intracortical gap opposite the injection site at POW 16 (Fig. 5Bi). The predicted amount of soft tissues present in the callus in that case agreed well with the experimentally measured amount (Fig. 5Bii). The use of an injectable carrier to deliver the cells close to the fracture healing site did not generate a good healing response *in silico*. The limitation of the MSCs' migration speed due to the fibrous extracellular matrix that has formed during the first three weeks is not problematic in case of injection directly into the callus area. However, this does become an issue when the cells are administered in a carrier close to (but not within) the fracture site. MSCs entering the callus area start differentiating under the influence of the growth factors that are present. As the cells migrate slowly into the callus area, they become differentiated before they reach the central area of the callus. Differentiated cells deposit bone matrix to replace the fibrous matrix which further decreases the migration speed of the cells that enter the callus area while the osteogenic growth factors that are expressed enhance the differentiation, resulting in a layer of bone close to the cell source while fibrous tissue persist in the major part of the callus area.

In this study we have shown that a mathematical model, initially developed for normal fracture healing, can be used as a clinical tool to investigate aetiology and treatment of atrophic non-unions. Despite a number of deviations mainly due to simplifications in the model, the mathematical model is able to capture essential aspects of the atrophic non-union as observed experimentally *in vivo*. Interestingly, the correspondence between simulations and experiments was obtained without changing the previously established parameter values, which clearly adds to the model's potential. Moreover, the model can be used to design treatment strategies, assist in the interpretation of experimental observations and test *in silico* various hypotheses in order to explain unexpected experimental results. Following such a combined *in silico*-*in vivo* approach may help to optimize experimental and clinical studies in this area.

## Supporting Information

Figure S1 Growth factor expression in healing and non-union groups. Immunohistochemical analysis of TGF-beta, FGF-b, PDGF and BMP2/4 in healing and non-union groups at 1 and 3 weeks post-osteotomy. Images taken at ×20 or ×40 magnification and positive growth factor staining is seen as a red signal.
(6.59 MB TIF)

Text S1 This file contains a detailed description of the mathematical model used in this study including the full set of equations, parameter values, boundary & initial conditions and implementation details.
(0.67 MB DOC)
