# Supplementary figures and images for: Occurrence and Treatment of Bone Atrophic Non-Unions Investigated by an Integrative Approach
Source: PLoS Comput Biol. 2010 Sep 2;6(9):e1000915. doi: 10.1371/journal.pcbi.1000915 (PMC2932678; doi:10.1371/journal.pcbi.1000915)

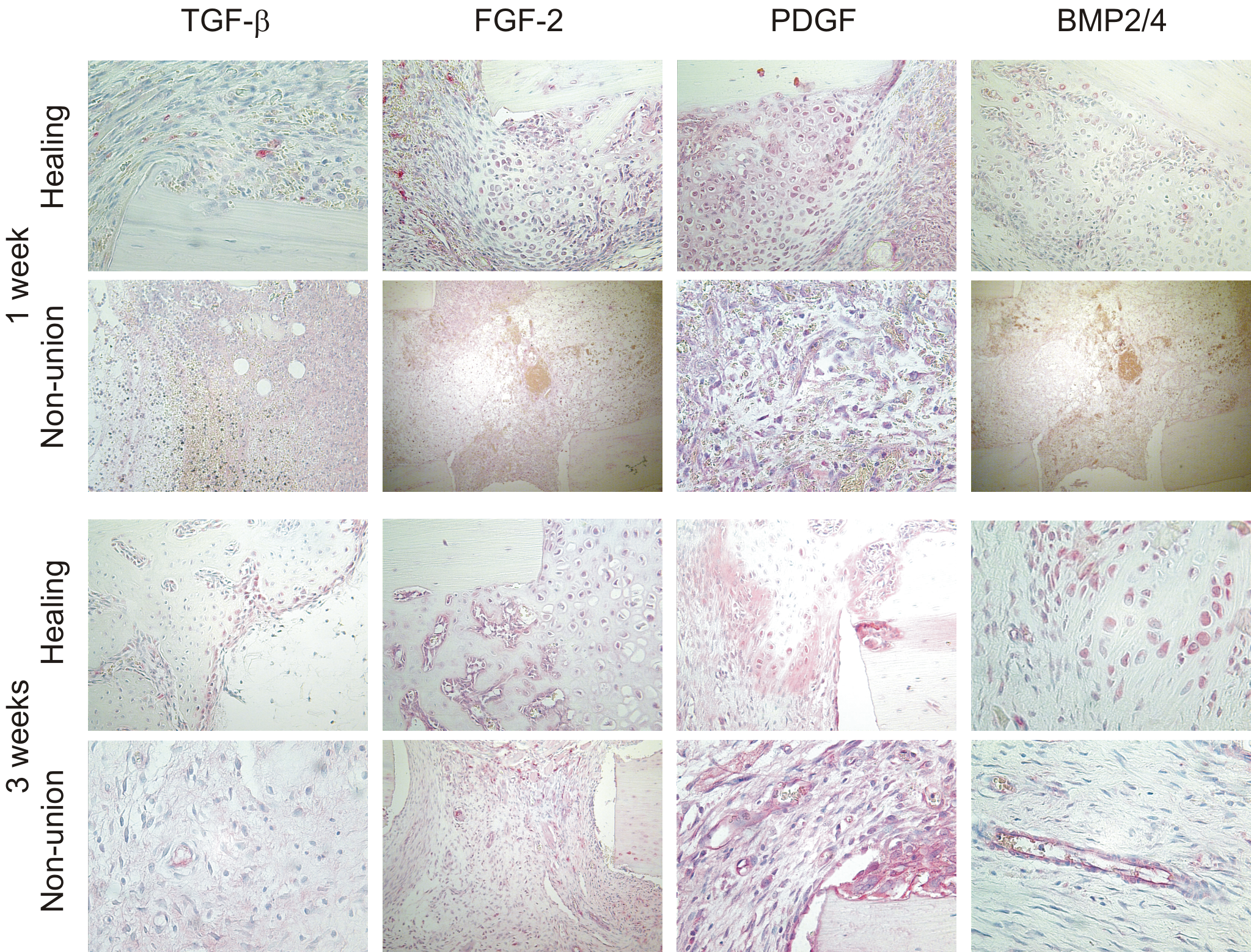

Supplement: Figure S1 — Growth factor expression in healing and non-union groups. Immunohistochemical analysis of TGF-beta, FGF-b, PDGF and BMP2/4 in healing and non-union groups at 1 and 3 weeks post-osteotomy. Images taken at ×20 or ×40 magnification and positive growth factor staining is seen as a red signal. (6.59 MB TIF) [file pcbi.1000915.s001.tif]
