# Supplementary material for: Occurrence and Treatment of Bone Atrophic Non-Unions Investigated by an Integrative Approach
Source: PLoS Comput Biol. 2010 Sep 2;6(9):e1000915. doi: 10.1371/journal.pcbi.1000915 (PMC2932678; doi:10.1371/journal.pcbi.1000915)
Supplement: Text S1 — This file contains a detailed description of the mathematical model used in this study including the full set of equations, parameter values, boundary & initial conditions and implementation details. (0.67 MB DOC) [file pcbi.1000915.s002.doc]

**Supplementary methods**

***Mathematical model***

The following equations describe the spatiotemporal evolution of the densities of mesenchymal stem cells (c_m_), fibroblasts (c_f_), chondrocytes (c_c_), osteoblasts (c_b_), endothelial cells (c_v_), fibrous tissue (m_f_), cartilage (m_c_), bone (m_b_) and vascular matrix (m_v_) and the concentrations of 3 generic growth factor families, chondrogenic (g_c_), osteogenic (g_b_) and vascular growth factors (g_v_) constituting the model applied in this study:

Following functional forms describe the nonlinear and variable dependent nature of diffusion, taxis, proliferation, differentiation and growth factor production rates:

The following scalings were chosen for the non-dimensionalisation of the variables:

Typical time and length scales for fracture healing in rodent studies are T = 1 day and L = 3.5 mm (Harrison et al., 2003). A representative concentration of the collagen content in the tissues under investigation is m_0_ = 0.1 g/ml. Typical growth factor concentrations are in the order of magnitude of 10^-9^ M (mol/l) (Nogami and Oohira, 1984; Joyce et al., 1990). Taking into account the order of magnitude of the molecular weight of the growth factors (100 kDa = 100 kg/mol), this results in a non-dimensionalisation value of g_0_ = 100 ng/ml. Based on geometrical constraints, Bailón-Plaza and van der Meulen (2001) derived a typical value for cell density at the beginning of the healing process: c_0_ = 10^6^ cells/ml.

The parameter values were derived from literature where possible and estimated when no relevant data was available. We refer to Geris et al. (2008) for a detailed description of the parameter derivation and estimation. The parameters were non-dimensionalised as follows (tildes referring to non-dimensionalised values):

This resulted in the following set of non-dimensional parameter values:

The system of equations must be complemented by suitable initial and boundary conditions to ensure the existence, uniqueness and non-negativity of a solution (schematically indicated in Fig. 2b of the main manuscript text). At the start of the simulation, the entire callus area was filled with a loose fibrinous tissue matrix (
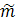
 = 0.1). Resulting from the haemorrhage, vascular growth factor was present throughout the callus (
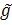
f_ini = 10). All other variables were assumed to be zero initially. The mathematical model was closed by prescribing suitable boundary conditions. No-flux boundary conditions were applied for all variables carrying diffusion or taxis terms in their equations, except for the situations described below, where Dirichlet boundary conditions (i.e. concentration assigned at the boundary mimicking presence of that variable outside of the simulation domain) were prescribed for certain components on specific parts of the boundary and for a specified period of time.

Mesenchymal stem cells and fibroblasts were released into the callus tissue at the beginning of the healing process from three possible sources: the periosteum, the surrounding soft tissues and the marrow space at the site of the damaged cortical tissue (Gerstenfeld et al., 2003). The experimental set-up for normal healing simulated in this study did not compromise any of these sources (in contrast to the nonunion group, as discussed in the main manuscript text), therefore all sources were adopted here (
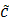
m_bc = 0.02 &
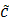
f_bc = 0.02 during first 3 days). The intact vasculature in the cortical bone and marrow cavity were modelled as a source of endothelial cells throughout the entire regeneration process (
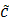
v_bc = 10^-4^) (Gerstenfeld et al., 2003a). Chondrogenic and osteogenic growth factors were assumed to originate from respectively the fractured bone ends and the cortex away from the fracture site (Barnes et al., 1999; Dimitriou et al., 2005) (
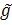
c_bc =
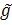
b_bc = 20 during respectively 5 and 10 days).

***Implementation***

The 12 variables are non-negative. This qualitative property of the solution must be inherited by its numerically computed approximation because, amongst others, erroneous negative values for the concentrations might render otherwise stable reaction terms unstable. Besides ensuring non-negativity, the algorithm employed for the numerical solution of the model must respect conservation of mass. The finite volumes technique was employed for its inherent mass conservation properties. The Method of Lines (MOL) was applied to separate the spatial and temporal discretisation (Hundsdorfer and Verwer, 2003). The axi-symmetric structure of the problem was employed to reduce the model to an equivalent problem in 2D space, leading subsequently to an efficient spatial discretisation (Gerisch and Geris, 2007).

The spatial domain was covered with an equidistant computational grid. After convergence tests the grid size was fixed at 0.05 mm in both directions. On this grid, the diffusion and reaction terms in the system of equations (1-12) were discretised using respectively the standard second order central difference approximation and pointwise evaluation, which were found to be sufficient in terms of accuracy and to ensure non-negativity of the solution of the resulting ordinary differential equation (ODE) system. Contrarily, the discretisation of the taxis terms in this system of equations required the application of upwinding techniques with nonlinear limiter functions (van Leer limiter) to guarantee accurate, non-negative solutions of the MOL-ODE system (Gerisch and Chaplain, 2006). The order of the spatial approximation is two in general. For the time integration of the resulting stiff MOL-ODE system the code ROWMAP (Weiner et al., 1997) was used. The methods built-in automatic step size control ensures the error caused in each time step (local error) to remain below a user-prescribed tolerance while keeping the computational cost as low as possible.

For a discussion of the simplifications related to the methods described above, we refer the reader to Geris et al. (2008).

***References of the supplementary methods***

Bailón-Plaza, A., van der Meulen, M.C.H. A mathematical framework to study the effects of growth factor influences on fracture healing. *J. Theor. Biol.* **212**, 191-209 (2001).

Barnes, G.L., Kostenuik, P.J., Gerstenfeld, L.C., Einhorn, T.A. Growth factor regulation of fracture repair. *J. Bone Miner. Res.* **14**(11), 1805-1815 (1999).

Dimitriou R., Tsiridis, E., Giannoudis, P.V. Current concepts of molecular aspects of bone healing. *Injury* **36**(12), 1392-1404 (2005).

Geris, L., Gerisch, A., Vander Sloten, J.V., Weiner, R., Van Oosterwyck, H.V. Angiogenesis in bone fracture healing: a bioregulatory model. *J Theor Biol.* **251,** 137-58 (2008).

Gerisch, A. & Chaplain, M.A.J. Robust numerical methods for taxis-diffusion-reaction systems: Applications to biomedical problems. *Math. Comput. Model.* **43**, 49-75 (2006).

Gerisch A. and Geris L. A finite volume spatial discretisation for taxis-diffusion-reaction systems with axi-symmetry: application to fracture healing. In: Deutsch, A., Brusch, L., Byrne, H., de Vries, G., Herzel, H.-P. (Eds.), *Advances in Mathematical Modeling of Biological Systems, Volume I,* Birkhäuser, Boston, ISBN 0-8176-4557-8, 303-316 (2007),

Gerstenfeld, L.C., Cullinane, D.M., Barnes, G.L., Graves, D.T., Einhorn, T.A. Fracture healing as a post-natal developmental process: molecular, spatial, and temporal aspects of its regulation. *J. Cell. Biochem.* **88**, 873-884 (2003).

Harrison, L.J., Cunningham, J.L., Strömberg, L., Goodship, A.E. Controlled induction of a pseudarthrosis: a sudy using a rodent model. *J. Orthop. Trauma* **17**, 11-21 (2003).

Hundsdorfer, W., Verwer, J.G. *Numerical Solution of Time-Dependent Advection-Diffusion-Reaction Equations.* Springer Series in Computational Mathematics 33, Springer Verlag (2003).

Joyce, M.E., Terek, R.M., Jingushi, S., Bolander, M.E. Role of transforming growth factor-b in fracture repair. *Ann. N. Y. Acad. Sci.* **593**, 107-123 (1990).

Nogami, H., Oohira, A. Postnatal new bone formation. *Clin. Orthop. Relat. Res.* **184**, 106-113 (1984).

Weiner, R., Schmitt, B.A., Podhaisky, H. ROWMAP - a ROW-code with Krylov techniques for large stiff ODEs. *Appl. Numer. Math.* **25**, 303-319 (1997).
